# Synergistic effects of gamma-aminobutyric acid and melatonin on seed germination and cadmium tolerance in tomato

**DOI:** 10.1080/15592324.2023.2216001

**Published:** 2023-06-11

**Authors:** Yiying Lv, Yongteng Zhao, Yuansheng He, Jiming Wang, Yuanxian Zheng, Xiaolong Chen, Feiyan Huang, Jiani Liu, Lei Yu

**Affiliations:** a Yunnan Urban Agricultural Engineering and Technological Research Center, College of Agronomy, Kunming University, Kunming, China; b Faculty of Life Science and Technology, Kunming University of Science and Technology, Kunming, China; c Yunnan Tobacco Company Lincang Company, Lincang, Yunnan, China; d Henan China Tobacco Industry Co. Ltd, Zhengzhou, Henan, China

**Keywords:** Tomato, seed germination, cadmium stress, γ-aminobutyric acid, melatonin

## Abstract

The effects of exogenous γ-aminobutyric acid (GABA) and melatonin (MT) on tomato seed germination and shoot growth exposed to cadmium stress were investigated. On the one hand, treatment with MT (10–200 μM) or GABA (10–200 μM) alone could significantly relieve cadmium stress in tomato seedlings, which is reflected in increasing the germination rate, vigor index, fresh weight, dry weight and radicle lengths of tomato seeds, as well as the soluble content compared to the absence of exogenous treatment, and the alleviating effect reached the peak in the 200 µM GABA or 150 µM MT alone. On the other hand, exogenous MT and GABA showed synergistic effects on the germination of tomato seed under cadmium stress. Moreover, the application of 100 µM GABA combined with 100 µM MT markedly decreased the contents of Cd and MDA by upregulating the activities of antioxidant enzymes, thereby alleviating the toxic effect of cadmium stress on tomato seeds. Collectively, the combinational strategy showed significant positive effects on seed germination and cadmium stress resistance in tomato.

## Introduction

Cadmium (Cd), an unnecessary but highly toxic heavy metal element for living organisms, is increasing in agricultural soils as a result of industrial, agricultural and urban activities. Cd can rapidly be absorbed by plant roots and enriched in different tissues due to its high soil mobility and strong plant invasiveness^1^. Cd accumulation inhibits plant respiration and photosynthesis, impairs the activity of antioxidant enzymes, causes physiological and biochemical disorders in plants, and leads to disruption of basic metabolic functions that affect plant yield, quality, and safety^2,3^. Furthermore, Cd can accumulate in the edible parts of plants, which further enters into human body through food chain to damage immune system, urinary system, and nervous system^4^. Thus, it is essential to develop reliable methods to mitigate Cd phytotoxicity and reduce Cd uptake in plants.

Plants have evolved many complex mechanisms to regulate cadmium uptake and minimize its hazards^5^. First, some carbohydrates exist in the cell wall and extracellular carbohydrates, such as amino acids, ferritins and organic acids, can reduce the Cd content in the plant by immobilizing Cd^6^. The plasma membrane is the second barrier hindering cadmium adsorption. Cd causes the oxidation and cross-linking of protein thiols, or inhibits key membrane proteins such as H^+^-ATPase, or alters the composition and fluidity of membrane lipids, further damaging other organelles^7^. Third, the presence of some organelles may hinder the diffusion of cadmium ions; for example, the compartmentalization of cadmium in vacuoles attenuates the flow of cadmium ions in the cytosol, forcing these ions into a limited area, thus reducing the widespread binding toxicity of cadmium ions to the organelles^8^. Finally, when plants are exposed to Cd, some general biochemical stress defense reactions are initiated to reduce cadmium toxicity^9^, such as the induction of antioxidant enzymes. In addition, many studies have shown that antioxidant defense systems are strengthened in a number of plant species by introducing signaling molecules^10,11^. Dawuda et al^12^ found that abscisic acid could alleviate the toxic effects of Cd on plants by increasing antioxidant enzyme activity and photosynthesis and reducing Cd levels in lettuce plants. Similarly, salicylic acid (SA) was shown to enhance the tolerance of potato to Cd stress by increasing the relative water content and proline and enhancing the expression level of selected genes related to SA and reactive oxygen species (ROS) metabolism^13^. Furthermore, Amjadi et al^14^ found that MT combined with SA treatment more efficient than MT and SA singly in the improvement of detrimental effects induced by Cd toxicity. The same thing happens with salicylic acid and nitric oxide in alleviating zinc toxicity of Safflower (*Carthamus tinctorius* L.)^15^. Therefore, hormones coupled with other signal molecules may produce synergistic effects in regulating plant stress tolerance by regulating various signaling pathways.

The signaling molecule gamma aminobutyric acid (GABA) has been widely reported to play a vital role in plant stress resistance. It acts as an endogenous signaling molecule in plant growth and development, regulating the osmotic pressure balance of plants under stress in coordination with plant hormones, thereby improving plant stress resistance^16,17^. In addition, as a class of four-carbon nonprotein amino acids, it can regulate the two major metabolic pathways of carbon and nitrogen in plants (such as regulate carbon and nitrogen balance and cytoplasmic pH, promote the metabolism of amino acids and carbohydrates, and induce ethylene synthesis) and thus participate in the regulation of plant growth and development^18,19^.

Melatonin is a small molecule indoleamine compound also known as pineal hormone. It was originally thought to exist only in humans and animals, but subsequent studies have found that it is also present in various organs of vegetables, fruits and commercial crops^20,21^. Numerous studies have suggested that melatonin plays a crucial biological role in inducing seed germination, promoting root development and delaying senescence^22,23^. Moreover, melatonin also acts as a biostimulant that increases tolerance to biotic stress. When plants are subjected to stress, melatonin can reduce the accumulation of reactive oxygen species in plants and upregulate antioxidant enzyme activity to increases plant stress resistance^24–26^. Notably, numerous studies have shown that melatonin can regulate plant growth and stress responses through interactions with other phytohormones^27,28^. There have some documents addressing the interactive effects of GABA and MT, for instance, Sharafi et al.^29^ found that melatonin triggers GABA-shunt during stress responses in tomato. Treatment with GABA and MT have the synergistic effects on protection of photosynthesis system in response to abiotic stresses in tomato, such as polyethylene glycol and high salinity^30^. However, the interactive effects of GABA and MT on Cd tolerance in tomato have not yet been investigated. Furthermore, the differences among the GABA, MT and GABA plus MT in the regulation of oxidative stress-caused by cadmium toxicity have not been compared in tomato. Therefore, in this study, tomato was used as a model plant, and the effects of exogenous GABA, MT, and their combinations application on improving plants tolerance to Cd phytotoxicity were investigated.

## Materials and methods

### Plant materials

The study was conducted at the Research Center of Urban Characteristics Agricultural Engineering in Yunnan Province of Kunming University. Tomato seeds were provided by Jinsheng Seed Industry Co., Ltd. (Xi’an, China).

### Germination tests

The experiment was divided into three groups, MT, GABA, and GABA plus MT (Equal concentration one to one mixed), and 7 treatments were setup in each group: CK (control, distilled water only), Cd (100 µM Cd, without soaking seeds in plant growth regulator), 10 µM (100 µM Cd, with soaking seeds in plant growth regulator at a concentration of 10 µM), 50 µM (100 µM Cd, with soaking seeds in plant growth regulator at a concentration of 50 µM), 100 µM (100 µM Cd, with soaking seeds in plant growth regulator at a concentration of 100 µM), 150 µM (100 µM Cd, with soaking seeds in plant growth regulator at a concentration of 150 µM), 200 µM (100 µM Cd, with soaking seeds in plant growth regulator at a concentration of 200 µM) Each treatment was replicated three times. Uniform tomato seeds were selected, disinfected and soaked; rinsed with distilled water 3 times; placed on absorbent paper to absorb moisture on the surface of the seeds; soaked with different concentrations of plant growth regulator solutions at room temperature for 24 hours in the dark; and then placed on two layers of filter paper pre-moistened with 3 mL of deionized water in covered 9 cm diameter petri dishes. Finally, 10 mL of solutions of different Cd^2+^ concentrations prepared in advance with deionized water and hydrated cadmium chloride (CdCl_2_∙2.5 H_2_O) were added to the petri dishes. Then, covered the petri dish and placed it in a temperature-controlled incubator under a 16/8 light photoperiod at 24°C (day)/18°C (night). The light intensity was 6000 lx. During the cultivation period, seed germination was observed and recorded regularly every day, and the evaporated water was supplemented by the weighing method. After 7 days of treatment, phenotypes were recorded and sampled.

### Seed germination assessment

The seed germination number was recorded every day during the cultivation process, and the germination potential (GP) and germination rate (GR) were calculated on the 3rd and 7th days, respectively^31,32^. The length of a germ was more than half the length of the seed itself, which is the standard for seed germination^33^. The germination index (GI) and the vigor index (VI) were calculated by referring to Zhao et al.^34^, GI = ∑ (Gt/Tt), where Gt is the number of germinated seeds per day and Tt is the corresponding number of germination days. VI = GI × fresh weight (FW) of germinated seeds on day 7. Three tomato seedlings were randomly selected from each petri dish, the fresh weight, dry weight and radicle lengths were measured. The dry weight was determined after oven drying at 80°C to a constant weight.

### Physiological index determination

The leaf tissues of fresh tobacco after treatment were used for physiological index determination. The degree of membrane lipid peroxidation was assessed by measuring the amount of MDA produced by the MDA kit. The content of soluble protein was determined using the assay kit and was measured at 562 nm. POD and CAT activities were measured by using the corresponding kits and was measured at 470 nm and 240 nm, respectively. The above kits are provided by Suzhou Keming Biotechnology Co., Ltd. The cadmium content was assayed using the method developed by Zhao et al.^35^

### Data analysis

The data obtained were expressed as the mean ± standard deviation of three independent trials and analyzed by one-way ANOVA (SPSS 22.0). The multiple comparison least difference method was used to test for differences between groups in different trials. Differences were considered significant if *P* < 0.05.

## Results and discussion

### Effects of exogenous GABA and MT on tomato seed germination under Cd stress

**Figure 1.**
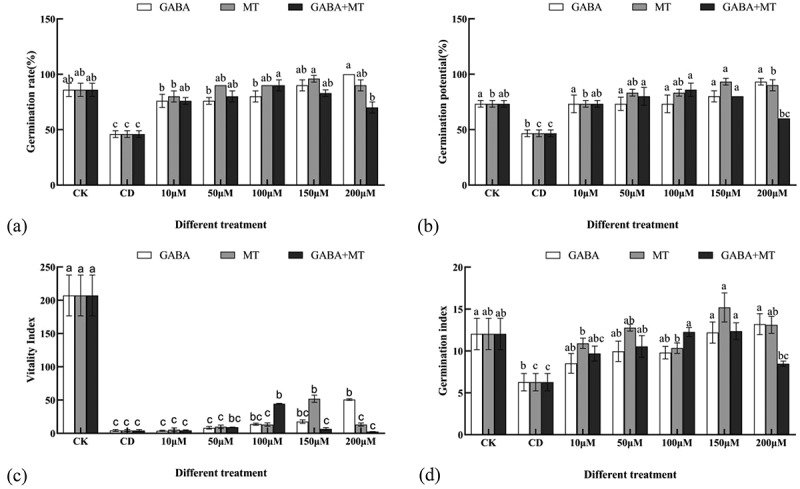
Effects of exogenous GABA and MT on tomato seed germination under Cd stress. (CK, the control; CD, 100 µM Cd; 10, 50, 100, 150, 200 µM, repent the tomato seedlings treatment with GABA, MT and GABA plus MT at 10, 50, 100, 150 and 200 μM, respectively, in the presence of 100 μM Cd. A: Germination rate experiment were calculated on 7th day after sowing; B: Germination potential experiment were calculated on the 3rd day after sowing; C: Vitality Index experiment were calculated on 7th day after sowing; D: Germination Index experiment were calculated on 7th day after sowing. The data shown are the averages of three replicates, with the standard errors indicated by the vertical bars. The means denoted by the same letter do not significantly differ at a P < 0.05.

The GR, GP, GI and VI significantly decreased in response to Cd stress (Figure 1). Cadmium stress affects the internal growth system of plant seeds^36^, which might reduce the germination of seeds. In this study, when the tomato seeds were exposed to 100 µM Cd, GR, GP, GI and VI of the seeds were significantly (*P* < 0.05) decreased compared with the control seeds; these indices were 53.49%, 63.71%, 52.05% and 2.10% of CK, respectively, indicating that seed germination of tomato was highly sensitive to cadmium (Figure 1). However, by using GABA or MT treatments (10–200 μM), these GR, GP, GI and VI of Cd-stressed tomato seedlings increased significantly, in comparison with those treated with Cd only (Figure 1). The GR, GP, GI and VI increased with increasing GABA concentration and reached the maximum at 200 µM in the GABA single treatment. In the MT single treatment, the GR, GP, GI and VI first increased and then decreased with increasing treatment concentration, which reached the peak value at 150 µM MT. The GR, GP and GI of tomato seeds could be significantly improved by GABA or MT alone treatment of 10–200 μM. But for VI, only the optimal concentration can be significantly improved. In GABA plus MT treatment, the GR, GP, GI and VI first increased and then decreased with increasing treatment concentration, which reached the peak value at 150 µM GABA. Moreover, the GABA or MT alone still kept a highly promotive role at 200 µM. In agreement with previous studies showing that MT regulation can improve seed germination under drought stress^37^. Cheng et al.^38^ showed that GABA activates amylase activity, accelerates starch catabolism, and produces metabolites such as soluble sugars, maintaining cell expansion and energy sources, thereby reducing adversity stress. However, a higher concentration of GABA inhibited seed germination of white clover under salt stress. This also occurs with melatonin^24^. These results suggest that the mitigating effect of MT and GABA on seed germination under stress conditions is closely related to their concentrations. More importantly, treatment with MT or GABA alone and their combination had positive role in the germination of tomato seeds under cadmium stress. However, the combinational treatment showed the phenomenon of “high concentration inhibition and low concentration promotion” in alleviating cadmium toxicity. Specifically, application of GABA plus MT significantly increased the levels of GR, GP, VI and GI compared with the cadmium stress alone at 10, 50 and 100 µM. Meanwhile, compared to the treatment with GABA or MT alone, the GI and VI of GABA plus MT-treated caused a sharp increase at 100 µM (Figure 1C-1D), while the GR and GP of GABA plus MT-treated doesn’t significantly (*P* < 0.05) enhanced (Figure 1A-1B). When the combinational treatment concentration was over 100 µM, the inhibitory effect of GABA plus MT treatment appeared by compared with that of the GABA or MT alone. These findings are consistent with previous studies showing that both GABA and MT have high concentration inhibitory effect. Lei et al^39^ showed that that MT pretreatment in the concentration-dependent manner significantly increased germination rate and promoted subsequent growth when wheat seeds exposed to Cr stress, and the mitigative effect could be counteracted at high concentration. Previous studies also found that high concentrations of melatonin inhibited seed germination^28^. When melatonin levels are above a certain threshold, ABA and indole-3-acetic acid (IAA) levels in *Arabidopsis* seeds increase, but GA levels reduce, which results in a decline in seed germination due to disordered hormone balance in seeds^28^. A plausible explanation could be excess ROS generation by the addition of high doses of these two regulators. Nevertheless, the specific mechanism remains to be further studied.

### Effects of exogenous GABA and MT on the growth of tomato seedlings under Cd stress

#### Sprout phenotype and biomass yield

**Figure 2.**
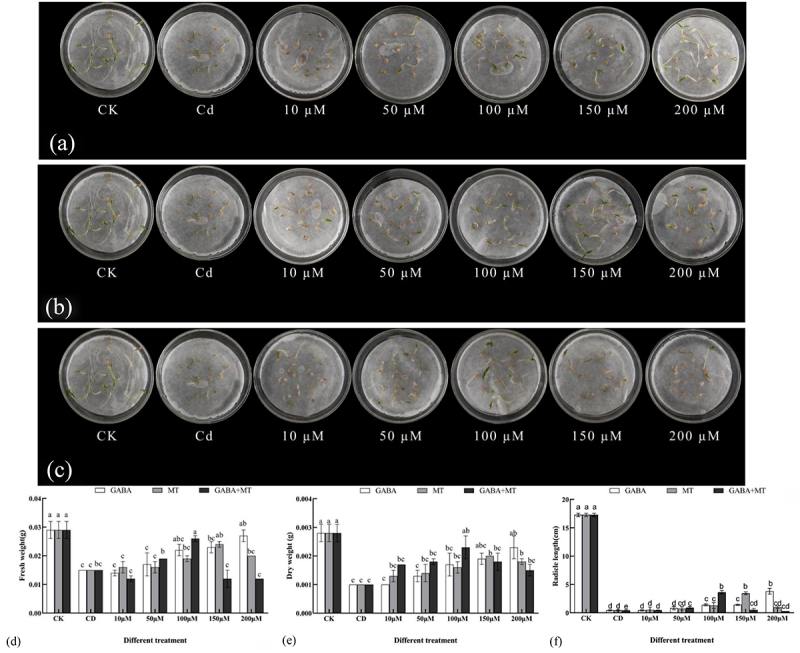
Effects of exogenous γ-aminobutyric acid, melatonin and their combination on the phenotype and biomass of tomato shoots under cadmium stress (CK, the control; CD, 100 µM Cd; 10, 50, 100, 150, 200 µM, repent the tomato seedlings treatment with GABA, MT and GABA plus MT at 10, 50, 100, 150 and 200 μM, respectively, in the presence of 100 μM Cd. A: MT-treated bud phenotype on 7th day after sowing; B: GABA-treated bud phenotype on 7th day after sowing; C: GABA plus MT-treated bud phenotype on 7th day after sowing; D: Effects of exogenous γ-aminobutyric acid and melatonin and their combination on fresh weight of tomato shoots under cadmium stress; E: exogenous γ-aminobutyric acid and melatonin and their combination on dry weight of tomato shoots under cadmium stress; F: exogenous γ-aminobutyric acid and melatonin and their combination on the radicle lengths of tomato shoots under cadmium stress). The data shown are the averages of three replicates, with the standard errors indicated by the vertical bars. The means denoted by the same letter do not significantly differ at a P < 0.05.

Plant growth traits are the most visual criterion to evaluate the growth status of the plant. Cadmium stress significantly inhibited the growth of tomato shoots, resulting in smaller and darker tomato seeds, yellowing of shoot roots and significantly lower biomass compared with controls (CK). Our results were in line with previous reports by Zhang et al.^40^, who indicated that the biomass yield sharply declined under Cd stress in tobacco. Compared with that of healthy plants, the fresh weight, dry weight and the radicle lengths under CD treatment decreased by 48.28%, 64.29% and 97.49%, respectively. By the application of GABA, MT and their combination, the biomass yield of the treatments varied at different concentrations. Plant growth under Cd stress increased remarkably (*P* < 0.05) with respect to seedlings grown under Cd alone (Figure 2). It is worth mentioning that there were differences in biomass in tomatoes treated with different concentrations of plant growth regulators. In the GABA alone treatment, the fresh weight, dry weight and the radicle lengths increased with the increasing GABA concentration. When the GABA concentration was increase to 200 µM, the dry weight, fresh weight and the radicle lengths significantly increased compared with the cadmium stress alone, while the radicle lengths significantly (*P* < 0.05) increased at 100, 150, 200 µM. In the MT alone treatment, MT (10–200 µM) significantly increased the levels of the fresh weight, dry weight and the radicle lengths compared with the cadmium stress alone. The radicle lengths significantly increased at 100 and 150 µM while the dry weight, fresh weight significantly increased only at 200 µM. Differently, in their combination treatment, the dry weight, fresh weight and the radicle lengths showed a trend of “first increase and then decrease” and reached the maximum value at 100 µM, When the combinational treatment concentration was over 100 µM, the inhibitory effect of GABA plus MT treatment appeared. Meanwhile, compared to the treatment with GABA or MT alone, the dry weight, fresh weight and the radicle lengths caused a sharp increase at 100 µM (Figure 2D-2F), This result suggests an interactive effect for GABA and melatonin. In accordance, enhancing the effect of melatonin on GABA homeostasis through triggering GABA-shunt pathway has been reported^29^. In this study, higher biomass production was obtained by GABA, MT and their combination compared to the biomass of the control. The concentration of 200 µM GABA or 150 µM MT had the best mitigation effect in the single-dosed treatment, while their combination that 100 µM GABA plus 100 µM MT is the best to relieve cadmium toxicity. Moreover, the synergistic effect was significantly demonstrated at the application of 100 µM GABA combined with 100 µM MT. It was reported that GABA increased biomass of microalgae under cadmium stress ^35^. Increase in biomass production was reported in wheat under Cd stress that were supplemented with melatonin^41^. Moreover, Amjadi et al.^14^ found that exogenous melatonin and salicylic acid had potentiating effects in alleviating cadmium stress in safflower seedlings, which is also reflected in MT and GABA treatments.

### Effects of exogenous GABA and MT on the antioxidant properties of tomato seeds under Cd stress

**Figure 3.**
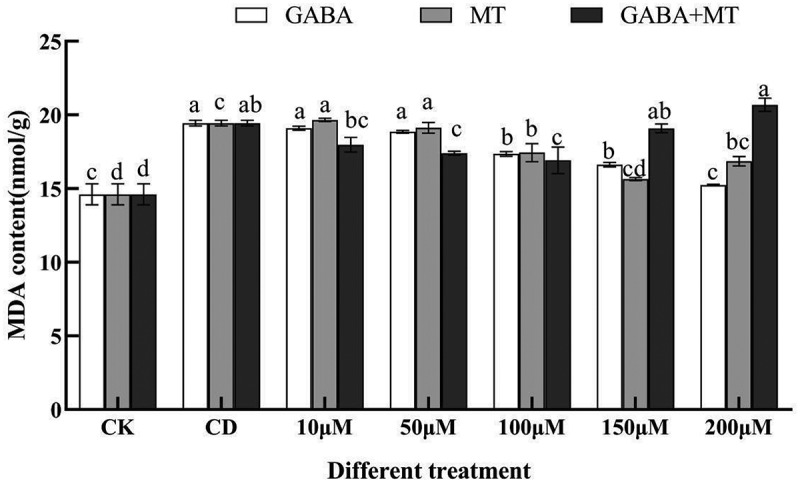
Effects of exogenous γ-aminobutyric acid, melatonin and their combination on the MDA content of tomato shoots under cadmium stress. (CK, the control; CD, 100 µM Cd; 10, 50, 100, 150, 200 µM, repent the tomato seedlings treatment with GABA, MT and GABA plus MT at 10, 50, 100, 150 and 200 μM, respectively, in the presence of 100 μM Cd). The data shown are the averages of three replicates, with the standard errors indicated by the vertical bars. The means denoted by the same letter do not significantly differ at a P < 0.05.

**Figure 4.**
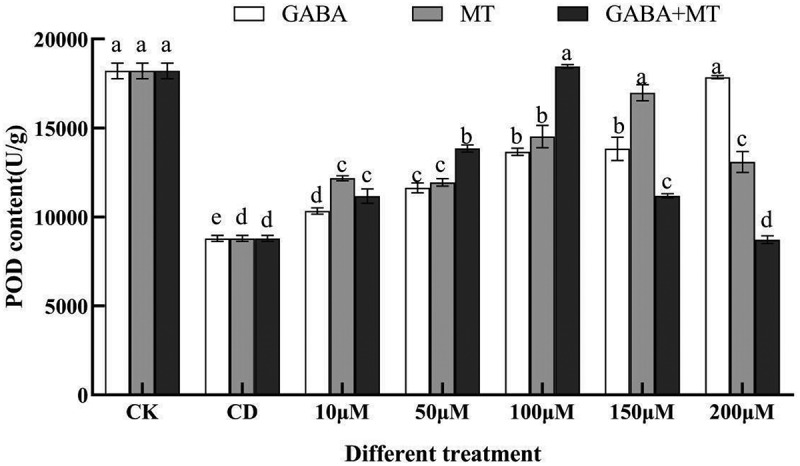
Effects of exogenous γ-aminobutyric acid, melatonin and their combination on the POD content of tomato shoots under cadmium stress. (CK, the control; CD, 100 µM Cd; 10, 50, 100, 150, 200 µM, repent the tomato seedlings treatment with GABA, MT and GABA plus MT at 10, 50, 100, 150 and 200 μM, respectively, in the presence of 100 μM Cd). The data shown are the averages of three replicates, with the standard errors indicated by the vertical bars. The means denoted by the same letter do not significantly differ at a P < 0.05.

**Figure 5.**
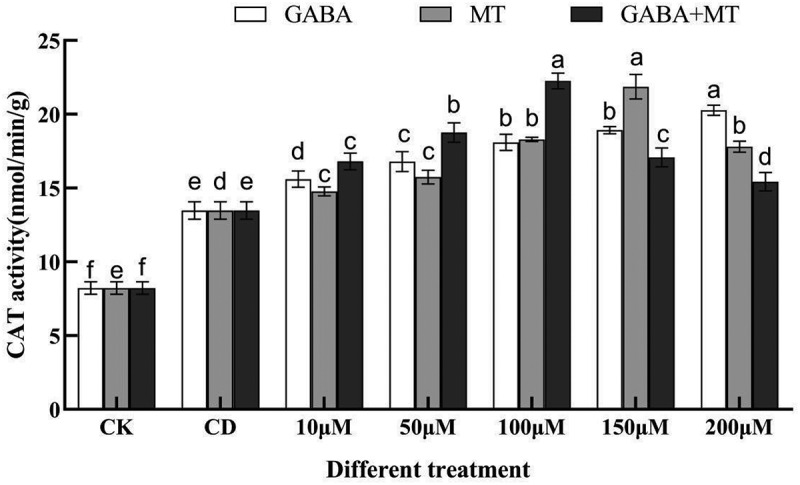
Effects of exogenous γ-aminobutyric acid, melatonin and their combination on the CAT activity of tomato shoots under cadmium stress. (CK, the control; CD, 100 µM Cd; 10, 50, 100, 150, 200 µM, repent the tomato seedlings treatment with GABA, MT and GABA plus MT at 10, 50, 100, 150 and 200 μM, respectively, in the presence of 100 μM Cd). The data shown are the averages of three replicates, with the standard errors indicated by the vertical bars. The means denoted by the same letter do not significantly differ at a P < 0.05.

Given that MT and GABA are well-known signaling molecules that enhance plant stress resistance^42,43^, determining whether these molecules could reduce MDA accumulation under Cd stress is important. The MDA content and several antioxidant activities enzymes (SOD, CAT) were compared between plant growth regulator-treated and untreated samples under 100 µM of Cd (Figures 3, 4 and 5).

MDA is one of the main products of membrane lipid peroxidation, and its accumulation reflects the degree of toxic damage caused by reactive oxygen species. The MDA contents significantly increased under Cd stress without plant growth regulator, while the MDA content under GABA, MT and their combination was significantly lower than that under the control. (Figure 3). Similarly, Nawaz et al.^44^ illustrated that the MDA contents in watermelon under vanadium stress with MT were lower than those in systems without MT. Moreover, it is worth mentioning that the MDA contents under cadmium treatment varied with plant growth regulator type and concentrations. The minimum MDA contents were observed at 200 µM (GABA treatment), 150 µM (MT treatment) and 100 µM (GABA plus MT treatment), which were 78.44%, 80.47% and 87.06% of that in the treatment of Cd alone, respectively. Exogenous GABA (10–200 µM) decreased the contents of MDA compared with the cadmium stress alone, among which 200 µM GABA significantly decreased MDA contents (Figure 3). Application of MT alone significantly decreased the accumulation of MDA compared with the cadmium stress alone at 150 and 200 µM. More importantly, treatment with GABA plus MT (10–100 µM) had positive role in reducing MDA accumulation in tomato under cadmium stress, whereas the negative effect was occurred at 150 and 200 µM. These data suggest that GABA, MT and their combination at appropriate dose can moderate MDA accumulation and reduce the degree of membrane lipid peroxidation under Cd stress.

The responses of antioxidant enzymes closely associated with plant adversity were analyzed in tomato (Figures 4 and 5). The POD activity was significantly (*P* < 0.05) enhanced at the concentration of 10 to 200 µM GABA compared with single cadmium stress and increased as the treatment concentration increased. Differently, the POD activity under the GABA plus MT treatment and the MT alone treatment first increased and then decreased, reaching the highest point at 100 µM and 150 µM, which was 2.10-fold and 1.93-fold higher than that of the single Cd treatment, respectively (Figure 4). For CAT activity, 100 µM of cadmium stress significantly (*P* < 0.05) increased the CAT activity of tomato, and GABA, MT and their combination also increased the CAT activity to a certain extent (Figure 5). Compared with single cadmium stress, GABA, MT and GABA plus MT treatments had the strongest CAT activity in 200 µM, 150 µM and 100 µM treatments, which were 150.45%, 162.26% and 165.16% of CD, respectively.

These results suggest that GABA, MT and their combination relieve cadmium toxicity by reducing the degree of membrane lipid peroxidation and enhancing antioxidant enzyme activity in tomato seedings. These findings are consistent with previous studies showing that exogenous MT can **reduced** MDA content and mitigation damage induced by abiotic stress in wheat seedlings^45^. Kumar et al.^43^ also reported that exogenous GABA (0.5 mM) application significantly reduced H_2_O_2_ and TBARS (Thiobarbituric acid reactive substances) levels and recovered growth parameters against As (III)-stressed rice seedlings. GABA reduced Cr uptake and upregulated nonenzymatic antioxidants and the activities of enzymatic antioxidants and finally reduced oxidative damage^46^. However, high concentrations of plant growth regulators can induce stress in plants, which adversely affects plant growth^47^. Treatment with 200 µM MT had higher MDA content and lower POD and CAT activities than those of 150 µM. The same situation was occurred in the combinational treatment with 150 and 200 µM compared with 100 µM. which might be due to the stress effect of excessively high concentration of regulators on tomato. Plant growth regulators are major determinants of the regulation of development and stress responses in plants, which can build the signaling networks with ROS to render an appropriate developmental and environmental response^48^. When the concentration of exogenous hormones is too high, imbalances between hormones and ROS can be caused, thus affecting cysteine-rich kinases (CRKs) which further damage cells by increasing sensitivity to abscisic acid (ABA) and osmotic stress^49,50^. Notably, compared to the treatment with GABA or MT alone, the POD and CAT activity under GABA plus MT caused a sharp increase at 100 µM (Figures 3, 4 and 5), and the MDA contents was decreased (Figures 3, 4 and 5). The synergistic activity is also reflected in response to high salinity stresses in tomato^30^. A plausible explanation could be GABA produced via MT promotion in GABA plus MT treatment, thus producing synergistic effect in improving the antioxidant capacity^51^. Collectively, suitable doses of MT and GABA had synergistic effect in alleviating oxidative damage in cadmium-stressed tomato seedlings through activation antioxidant enzymes.

### Effects of exogenous GABA and MT on the soluble protein of tomato shoots under Cd stress

**Figure 6.**
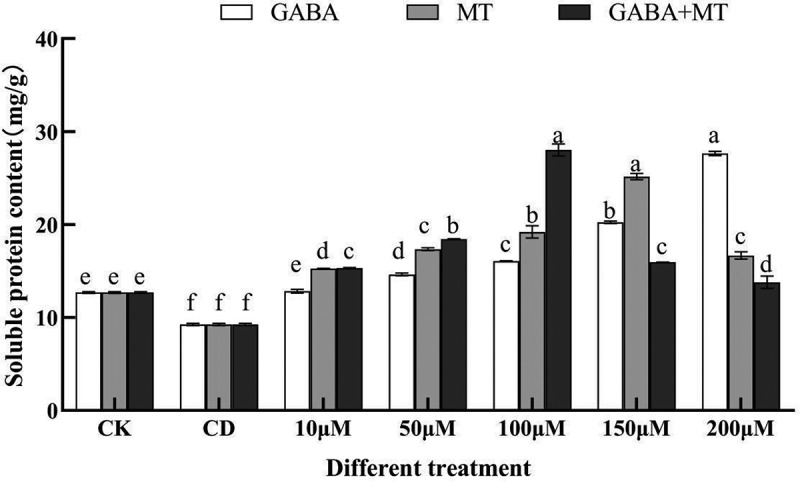
Effects of exogenous γ-aminobutyric acid, melatonin and their combination on the soluble protein content of tomato shoots under cadmium stress. (CK, the control; CD, 100 µM Cd; 10, 50, 100, 150, 200 µM, repent the tomato seedlings treatment with GABA, MT and GABA plus MT at 10, 50, 100, 150 and 200 μM, respectively, in the presence of 100 μM Cd). The data shown are the averages of three replicates, with the standard errors indicated by the vertical bars. The means denoted by the same letter do not significantly differ at a P < 0.05.

Soluble protein is an important nutrient that regulates cellular osmotic pressure and protects cell membranes and is generally regarded as one of the indicators for determining the osmoregulatory ability of plants. The soluble protein content of tomato shoots under 100 µM cadmium stress decreased by 27.13% compared with the control (CK) (Figure 6). The content of soluble protein was significantly increased (*P* < 0.05) after plant growth regulator treatment, and there were differences in the increase in soluble protein content with different concentrations of plant growth regulators. Compared with that of the CD control, soluble protein content in the GABA treatments (10, 50, 100, 150, 200 µM) increased by 38.74%, 58.17%, 73.66%, 118.91%, 198.90%, respectively; in the MT treatments it increased by 65.07%, 87.54%, 107.70%, 172.08% and 80.26%, respectively; in the GABA plus MT treatments, it increased by 65.55%, 99.45%, 203.13%, 72.52% and 49.13%, respectively.

When plants are exposed to adversity, tissue cells will accumulate osmoregulatory substances to resist the adversity to ensure normal physiological activities of plants. For example, Zhou et al.^52^ pointed out that the soluble protein and soluble sugar contents in camphor trees increased significantly under cadmium contamination, and the cellular water content was maintained at a reasonable level by enhancing the cellular water intake capacity, thus enhancing plant resilience, which is consistent with the results of this study. Moreover, compared to the treatment with GABA or MT alone, a synergistic effect was achieved under GABA plus MT at 100 µM (Figure 6). This may be due to the involvement of melatonin in mediation of GABA-shunt^29^ or inductive effect of melatonin on endogenous GABA^51^. Meanwhile, plant growth regulator treatment can upregulate osmoregulatory substances^53^. Some studies have also found that MT and GABA can resist some abiotic stresses such as heavy metal stress, cold stress by accumulating osmoregulatory substances^39,54^. However, the contents of soluble protein were lower at 150 μM and 200 μM treated with GABA plus MT than the GABA or MT alone, this phenomenon may be due to excess ROS generation by the addition of high doses of these two regulators disrupts the basic metabolism of the stress system, which involve synthesis of many proteins, particularly osmotin and osmotin-like proteins^55^.

### Effects of exogenous GABA and MT on cadmium content in tomato shoots under Cd stress

**Figure 7.**
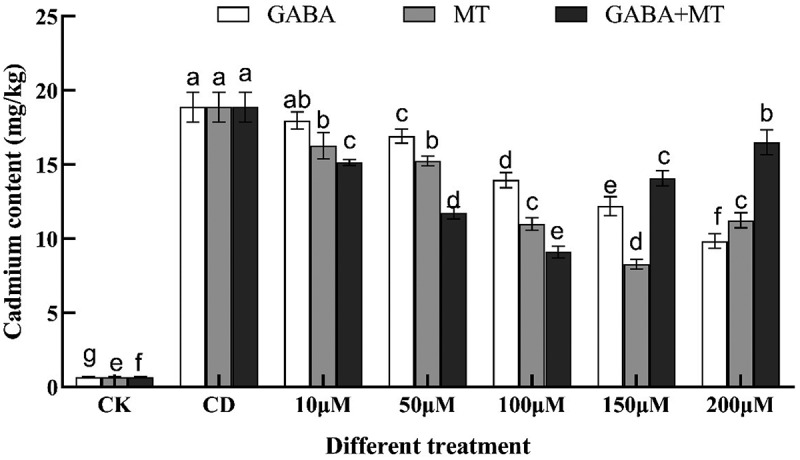
Effects of exogenous γ-aminobutyric acid, melatonin and their combination on Cd content of tomato shoots under cadmium stress. (CK, the control; CD, 100 µM Cd; 10, 50, 100, 150, 200 µM, repent the tomato seedlings treatment with GABA, MT and GABA plus MT at 10, 50, 100, 150 and 200 μM, respectively, in the presence of 100 μM Cd). The data shown are the averages of three replicates, with the standard errors indicated by the vertical bars. The means denoted by the same letter do not significantly differ at a P < 0.05.

Excessive accumulation of cadmium in plants interferes with the absorption and transportation of nutrients and threatens human health^56^. The present study found that single cadmium stress (100 µM) significantly increased the cadmium content of tomato shoots compared with no cadmium addition (CK) by 27.95-fold (Figure 7). The addition of MT and GABA reduced the cadmium content in the plants. It was reported that GABA reduced the uptake of Cd by reducing the net Cd^2+^ flux of apple root tips under Cd stress and the expression of genes related to Cd uptake and transport^41^. Decrease in cadmium content was reported in wheat under Cd stress that were supplemented with melatonin^41^ and in mallow that were primed with melatonin^57^. Moreover, application of GABA plus MT significantly reduced the uptake of Cd compared with the cadmium stress alone at 10, 50 and 100 µM. This suggests an interactive effect between GABA and melatonin on inhibiting cadmium absorption. And the combinational treatment concentration was increased at150 µM and 200 µM compared with that of 100 µM, which may be due to the stressful effect of excessive treatment concentrations on tomato and accelerated the disruption of the balance of its own metabolic system and caused irreversible damage to the plant, thus reducing its inhibition intensity.

## Conclusion

Our findings show that cadmium stress (100 μM) significantly inhibited the growth of tomato, while exogenous GABA, MT and their combination between 100 and 200 concentrations treatment can improve the GR and GP of tomato, enhance the seed VI and GI, and reduce the Cd content in tomato sprouts. Application of GABA, MT and their combination satisfactorily diminished the oxidative stress markers by upregulating antioxidant enzyme activities, increased soluble protein content and decreased cadmium content, thus enhancing cadmium stress resistance of tomato. Moreover, the application of 100 µM GABA combined with 100 µM MT showed the best effect on alleviating cadmium toxicity in tomato seedlings, which suggest that the GABA and MT at appropriate dose had synergistic effect on cadmium tolerance. Our findings provide a potential method for seed germination in response to cadmium toxicity and a theoretical basis for further exploration of the functions of exogenous plant growth regulators in alleviating cadmium stress in plants.
